# Organoid factory: The recent role of the human induced pluripotent stem cells (hiPSCs) in precision medicine

**DOI:** 10.3389/fcell.2022.1059579

**Published:** 2023-01-09

**Authors:** Giuseppe Novelli, Paola Spitalieri, Michela Murdocca, Eleonora Centanini, Federica Sangiuolo

**Affiliations:** ^1^ Department of Biomedicine and Prevention, University of Rome Tor Vergata, Rome, Italy; ^2^ IRCCS Neuromed, Pozzilli, IS, Italy; ^3^ Department of Pharmacology, School of Medicine, University of Nevada, Reno, NV, United States; ^4^ Department of Pharmacy, Health and Nutritional Sciences, University of Calabria, Rende, CS, Italy

**Keywords:** organoid factory, disease modeling., precision medicine, organoids manufacturing, human induced pluripotent stem cell (hiPSC)

## Abstract

During the last decades, hiPSC-derived organoids have been extensively studied and used as *in vitro* models for several applications among which research studies. They can be considered as organ and tissue prototypes, especially for those difficult to obtain. Moreover, several diseases can be accurately modeled and studied. Hence, patient-derived organoids (PDOs) can be used to predict individual drug responses, thus paving the way toward personalized medicine. Lastly, by applying tissue engineering and 3D printing techniques, organoids could be used in the future to replace or regenerate damaged tissue. In this review, we will focus on hiPSC-derived 3D cultures and their ability to model human diseases with an in-depth analysis of gene editing applications, as well as tumor models. Furthermore, we will highlight the state-of-the-art of organoid facilities that around the world offer know-how and services. This is an increasing trend that shed the light on the need of bridging the publicand the private sector. Hence, in the context of drug discovery, Organoid Factories can offer biobanks of validated 3D organoid models that can be used in collaboration with pharmaceutical companies to speed up the drug screening process. Finally, we will discuss the limitations and the future development that will lead hiPSC-derived technology from bench to bedside, toward personalized medicine, such as maturity, organoid interconnections, costs, reproducibility and standardization, and ethics. hiPSC-derived organoid technology is now passing from a proof-of-principle to real applications in the clinic, also thanks to the applicability of techniques, such as CRISPR/Cas9 genome editing system, material engineering for the scaffolds, or microfluidic systems. The benefits will have a crucial role in the advance of both basic biological and translational research, particularly in the pharmacological field and drug development. In fact, in the near future, 3D organoids will guide the clinical decision-making process, having validated patient-specific drug screening platforms. This is particularly important in the context of rare genetic diseases or when testing cancer treatments that could in principle have severe side effects. Therefore, this technology has enabled the advancement of personalized medicine in a way never seen before.

## 1 Introduction

Recently, the field of pluripotent stem cells (PSCs) has gained more and more attention as, over the years, it led to new findings on both human development and diseases. Novel molecular pathways and therapeutic targets were discovered in the past decades thanks to the research conducted culturing 2D stem cells, even though a further step forward has been made with the advent of 3D culture systems, as they allow and facilitate the simulation of *in vivo* conditions. Organoids are three-dimensional (3D) cell aggregates that accurately recapitulate the *in vivo* developmental stages of most organs and tissues.

The derivation process of PSC organoids exploits both the ability of these stem cells to differentiate into several cell types and the organ development process, including spatial patterning and morphogenesis (Clevers 2016; Eiraku, M. et al., 2011; Lancaster et al., 2013).

They are considered by the scientific community one of the biggest revolutions in the biomedical field. In fact, they were awarded method of the year in 2017 by Nature, and listed among the “fifty ideas that will change the world” by the Financial Times (Financial Times, 2022) and, according to Forbes, they were one of the five biggest innovations in oncology in 2019 (Five Things To Look Out For In Cancer Research In 2019; Forbes, 2022).

Organoids have been derived from many mammalian tissues, both normal and tumor, from adult stem cells and from pluripotent stem cells, both embryonic and induced pluripotent stem cells (iPSCs). Tissue organoids can retain many of the cell types and much of the structure and function of the organ of origin. Organoids derived from pluripotent stem cells display increased complexity compared with organoids derived from adult stem cells. Their major advantages are the ability to differentiate and self-renew making them an *in vitro* model that can precisely mimic the *in vivo* pathophysiology. PSC-derived human organoids have proved to be very useful for studying organ development where their propensity to reproduce embryonic stages of development has allowed the replication of key steps in organogenesis such as spatial organization of the heart, the brain, the gastrointestinal tract and other organs (Rossi et al., 2019; Renner et al., 2017; Finkbeiner, S. R., et al., 2015; McCracken, K. W. et al., 2014).

The use of human organoids in disease modelling has led to the establishment of assays and models that assist with diagnosis, drug screening and personalized treatment.

Organoids were first developed in 1987 (Corrò et al., 2020), after two important scientific successes: the application of collagenase for tissue digestion in the 1950s and the isolation of Matrigel in the 1980s (Xia et al., 2019). Since then, their accuracy and reproducibility have rapidly increased and are now considered a valid substitute for the *gold standard* (2D). They showed all their potential during the current SARS-CoV-2 pandemic: not only lung organoids were used to study the interaction between virus and host cells and the replication machinery, but they were also used as drug screening platforms for the development of new therapies and vaccines (Han et al., 2021; Spitalieri et al., 2022).

**FIGURE 1:**
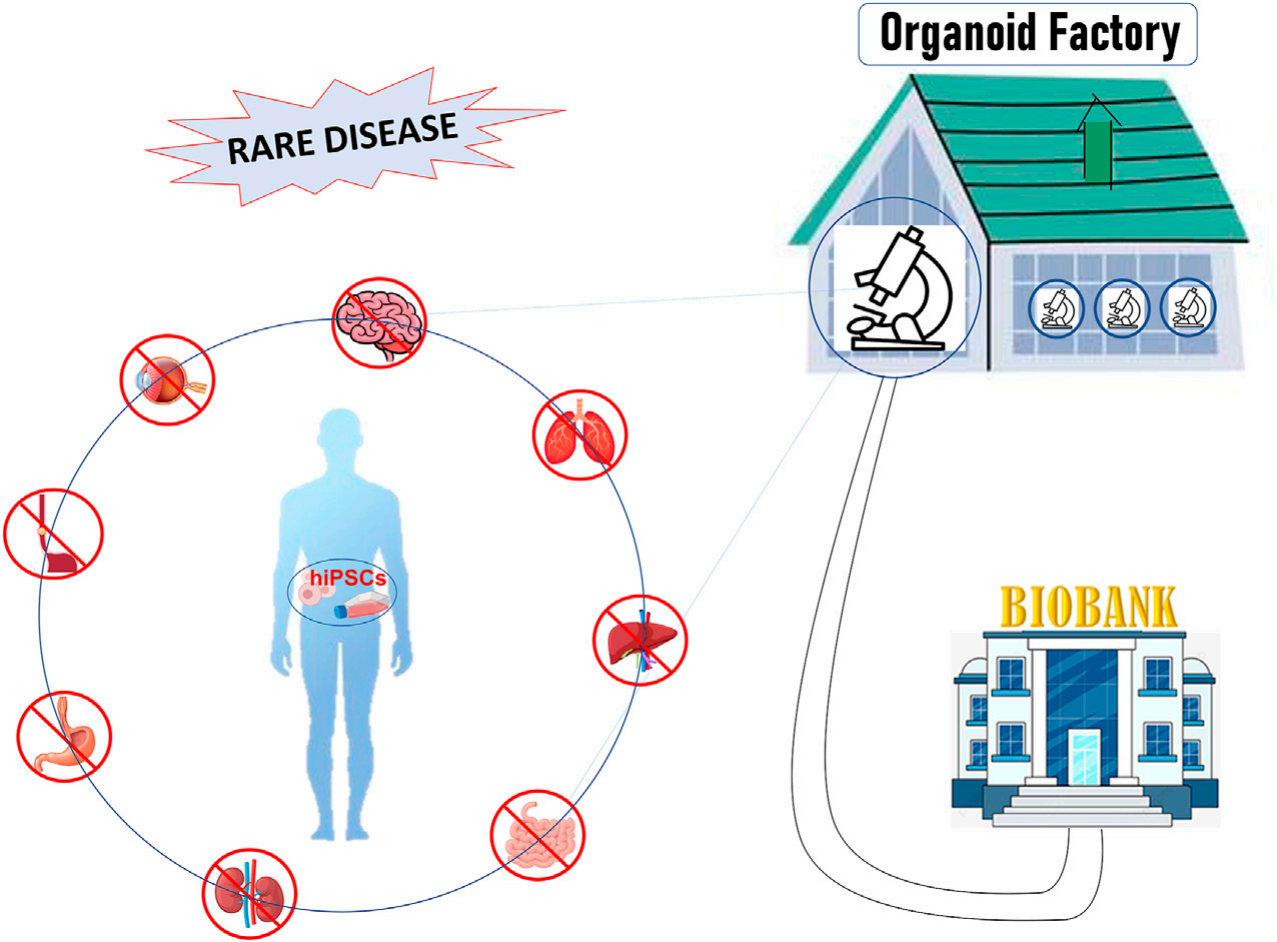
Figure 1: hiPSCs offer a unique patient-specific model to study rare disease: hiPSCs can be differentiated into organ-specific cells and assembled into organoids comprised of organ-specific tissue. Organoids factory offers the unique opportunity to create platforms for drug screening, regenerative medicine, toxicity profiling and biobanking, accelerating clinical decision-making process or the discovery of new therapies.

In this review, we will describe the main characteristics of 3D culture systems specifically derived from human induced pluripotent stem cells (hiPSCs), highlighting the state-of-the-art of organoid facilities that around the world offer know-how and service on organoid technology. Moreover, we will discuss the weaknesses and the future development that will lead this technology from bench to bedside, towards personalized medicine and biobanking (Figure 1).

## 2 Derivation and characteristics

The importance of 3D organoids lies in the following characteristics Fare clic o toccare qui per immettere il testo.

• Versatility: more than 14 kinds of organoids have been developed so far;
• Clinically relevant: they can be patient-specific and thus reproduce the physio-pathology of the organ *in vivo* keeping the same genotype of the patient;
• Genetically and phenotypically stable *in vitro* over long periods of time: they keep their characteristics when cultured and after cryopreservation;
• Expandible for large-scale analysis;
• Adaptable for gene editing techniques.

The organoids can be derived from different murine or human cell types: induced pluripotent stem cells (iPSCs) (Lancaster et al., 2013), adult stem cells (ASCs) (Sato et al., 2009), or embryonic stem cells (ESCs) (Jo et al., 2016). The latter is involved in embryonic development and has evident ethical problems that limit their applicability, while human iPSCs (hiPSCs) overcome this limitation as they are usually derived from a skin biopsy or other sources of adult cells and can be reprogrammed to be pluripotent stem cells, through the some reprogramming factors (*OCT4*, *KLF4*, *SOX2*, and *c-Myc*; OSKM) (Takahashi and Yamanaka, 2006). For these characteristics, hiPSCs are the most used type of cells for the derivation of organoids. In fact, hiPSCs can be differentiated into any kind of tissue by using specific growth factors. They will then self-assemble to form a complex structure and morphology, resembling the organ *in vivo*. Moreover, hiPSC-derived organoids are able to self-renew and be stable over a long period of time (Karagiannis et al., 2019). On the contrary, the major drawback of organoids derived from hASCs, organ-specific resident stem cells, is that they can be only composed of the cell types belonging to the original tissue, and consequently the development of these organoids is limited to the accessibility and availability of the hASCs (Mariano et al., 2015). To date, more than 14 types of organoids are reported in the literature and the majority of them are derived from hiPSCs. . Just to cite some of them, so far, the following kind of organoids have been developed: lungs (Han et al., 2021; Spitalieri et al., 2022), liver (Huang et al., 2022), gut (Sprangers et al., 2020), kidneys (van den Berg et al., 2020), brain (Agboola et al., 2021), retina (O’Hara-Wright and Gonzalez-Cordero, 2020), heart (Lee et al., 2022) and a lot more. For example, Van den Berg *et al.* (van den Berg et al., 2020) obtained glomerular, proximal, and distal tubular structures from hiPSC-derived kidney organoids. They tested the organoid efficacy *in vivo*, by transplanting it in immunodeficient mice. They observed that after vascularization, the glomerular filtration barrier was size-selective, giving evidence of the functionality of the produced organoid. Another interesting example of the complexity that organoids are able to recapitulate comes from heart models. Although there are studies on brain or vascular organoids reporting that they maintain their functionality through vascularization *in vivo* (Mansour et al., 2018; Wimmer et al., 2019), only Lee and collaborators demonstrated for the first time that human heart organoids has been implanted *in vivo* into nude mice and that beating is maintained through *in vivo* vascularization (Lee et al., 2022).

Indeed, heart organoids are still in their infancy and studies are investigating how to overcome the difficulties in mimicking the advanced structuring of the heart such as cardiac maturation and circulatory system and two-atrium/ventricles. It has been recently reported that 11 cardiac cell types directly constitute the heart, such as atrial cardiomyocytes, ventricular cardiomyocytes, fibroblasts, endothelial cells, pericytes, smooth muscle cells, immune cells (myeloid and lymphoid), adipocytes, mesothelial cells and neuronal cells (Litviňuková et al., 2020). Hence, it is clear that the heart can be considered one of the most difficult organs to model *in vitro*. Moreover, as the characteristics of previously reported heart organoids vary according to different differentiation methods, it is not yet possible to simulate all aspects of the heart. Some studies in this field are going in the direction of self-organizing heart organoids that can reproduce the complexity of the heart. Among them Lewis-Israeli, YR., et al. developed heart organoids that mimic the cardiac structure, reflecting the vascular network inside the organoid without external interference (Lewis-Israeli et al., 2021). Furthermore, Lee and collaborators demonstrated that in Chamber Formed-human Heart Organoids (CF-Hos) are able to spontaneously differentiate into cardiac-specific cells from hiPSCs without extrinsic interference (co-culture, 3D mold and physical stimulation, *etc.*) also showing the structural complexity of heart (chamber, atrium/ventricle-similar areas and epicardium/myocardium, *etc.*) *via* self-organization and the compartmentalization (Lee et al., 2022). Although the absence of endocardium (layer of endothelial cells) and the inability to construct a vascular network without the addition of ECM components, heart organoids reported by Lee reflect the advances on the field of health and welfare *via* cardiovascular modeling. Most recently, Branco and others developed a self-organized heart organoid containing an epicardium-like layer that entirely envelop a myocardium-like tissue enabling further insights into heart organogenesis and the development of myocardial-epicardial interaction (Branco et al., 2022). Finally, human iPSC-derived 3D organotypic cardiac microtissues have been generated and cultivated for more than 100 days, showing cardiac specification, survival and metabolic maturation. The authors demonstrated that this model could be used to study chemotherapy-induced cardiotoxicity (Ergir et al., 2022).

It is clear how the research in this emerging and expanding field is continuously making steps towards the clinical practice. Nevertheless, as noted above, there are some organoid models that are emerging in these years and need further developments, and others that already proved their functionality and utility, as the kidney organoid previously described.

Hence, the proven functionality of the organoids makes them suitable for studies aimed at testing new drugs, as they can fill the gap between the standard two-dimensional (2D) cell models and the animal models. While classic 2D *in vitro* models fail to represent the complexity of the organism as a whole, animal models have a different genome and pathophysiology than humans and not every disease has a corresponding validated animal model. Although to date, animal models are the *gold standard* and a mandatory step for drug screening tests and the translation of the compounds from the bench to the clinic, *in vitro* and *in vivo* tests in animals are still poorly predictive of the efficacy and safety in humans (Akhtar, 2015). Moreover, the sacrifice of animals for scientific purposes arises ethical concerns thus leading to the need of reducing the number of animals used, whenever possible, according to the 3 R’s principle: *replacement, reduction, and refinement*.

In the United Kingdom, the use of organoids as a drug screening platform led to an overall reduction in the number of animals used for scientific purposes by 7 percent annually in 2018 (Yaqub et al., 2022). Today, thanks to innovative technologies such as organoids, the transition from *in vivo* animal studies to *in vitro* analogs are facilitated, as 3D *in vitro* models are as complex and sometimes even more representative as in vivo human diseases.

The goal is to speed up the translation of research from the laboratories to the clinic and to reduce the number of animals needed for pre-clinical studies.

## 3 Applications

Organoids can be used for.

1. Basic research, including human biology studies aimed at understanding developmental processes, responses to external stimuli and stress signals, cell-cell interactions, and mechanisms of stem cell homeostasis;
2. As organ and tissue prototypes, especially for those difficult to obtain;
3. Drug screening and discovery, being a scalable and robust technology, to study the efficacy and side effects of a large number of molecules simultaneously and with great precision (Broutier et al., 2017; Medle et al., 2022)
4. Biobanking, as samples obtained from patients can be used to generate patient-derived organoids (PDOs) and stored as a resource for future research (Takebe et al., 2018; Jacob et al., 2020);
5. Disease modeling, to understand the mechanisms of human diseases such as infectious diseases, inherited genetic diseases, and cancer using multi-omics and drug screening analyses (Lancaster and Huch, 2019);
6. Precision medicine, in which patient-derived organoids can be used to predict drug response (Zhou et al., 2021a);
7. Tissue engineering, to replace or regenerate damaged tissue; organoids can be 3D printed, thus guiding the microscopic structure of the tissue (e.g., muscles) (Brassard et al., 2020);
8. Study of embryogenesis and developmental diseases (Kim et al., 2020).

## 4 Disease modeling

Originally, organoids were envisioned as an opportunity to grow healthy cells for applications in regenerative medicine and cell therapy. However, as knowledge about this new technology has advanced, much broader potential has been uncovered, ranging from diagnostic and drug screening to personalized medicine. In fact, organoids can be directly obtained from the patient’s own cells. In this way, a patient-specific model of the disease can be replicated *in vitro,* and treatment can be personalized. An overview of the hiPSCs-derived 3D organoids developed during the years and their relative applications on specific disease models can be explored in more detail in Table 1. According to the level of accuracy of the model, some of the organoids are used to gain new insights into the pathophysiology, while others are already applicable in the context of drug screening and discovery. Moreover, it can be pointed out that some diseases have systemic consequences, simultaneously influencing the physiology of several organs *in vivo*. Likewise, the same disease can be reproduced *in vitro* within different types of organoids. For example, it is the case of Cystic Fibrosis (CF), which is studied using with both an intestine and lung organoids (see Table 1).

**TABLE 1:** Examples of hiPSC-derived organoids used to model a specific disease (non-exhaustive list).

Organoid	*In vitro* disease model	References
Brain	ZIKA virus	Qian et al. (2016)
	Microcephaly	Lancaster et al. (2013)
	Autism	de Jong et al. (2021)
Lungs	Cryptosporidium	Heo et al. (2018)
	SARS-CoV-2	Spitalieri et al. (2022)
	Respiratory Syncytial Virus (RSV)	Harford et al. (2022)
	CF (*CFTR* gene)	Hirai et al. (2021)
Kidney	Polycystic kidney disease (*PKD* gene)	Freedman et al. (2015)
Eye	Retinite pigmentosa (*RPGR* gene)	Lane et al. (2020)
Esophagus	Barrett’s esophagus	(Zhang et al., 2022)*
Stomach	H-pylori	(Bartfeld et al., 2015)*
Intestine and Colon	Cryptosporidium	Heo et al. (2018)
	CF (*CFTR* gene)	(Vonk et al., 2020)*
	Intestinal Atresia (TTC7A gene)	(Bigorgne et al., 2014)*
	Microvillus inclusion disease (*STX3* gene)	(Wiegerinck et al., 2014)*
	Dyskeratosis congenital (*DKC1* gene)	(Woo et al., 2016)*
Liver	Alagille syndrome (*JAG1* gene)	(Shiota et al., 2021)*
	A1AT-deficiency	(Nuciforo and Heim, 2021)*
	Wolman’s disease	Ouchi et al. (2019)
	Steatosis	Ouchi et al. (2019)
	Primary sclerosing cholangitis	Soroka et al. (2019)
	Citrullinemia type I	Akbari et al. (2019)

The starred references (*) refer to organoids derived from cell sources other than hiPSC, such as hESCs, tissue specific stem cells, *etc.*

In the next subparagraphs, more detailed examples of how organoids are used in disease modeling will be illustrated. Although well-established organoids have already been generated for the brain, kidney, gut, lung and many other organs giving rise to disease models, organoid models of the heart have only begun to emerge in the last couple of years and, to our knowledge, papers with models of specific cardiac pathologies has not yet been published.

### 4.1 Infectious diseases

Organoids are increasingly used as a system to reproduce complex diseases *in vitro*. One of the advantages of using organoids for pre-clinical studies with respect to animal models is that, since they derive from human cells, they are not affected by interspecies differences. In addition, organoids can be genetically modified so that they express genes of interest that are useful, for example, to study host-pathogen interaction in the context of infectious diseases. Examples of this type of application are the study of the ZIKA virus on brain organoids and the use of intestinal organoids to study enteric viruses or the interaction between microbiota and intestinal epithelium (Artegiani and Clevers, 2018). More recently, lung organoids have been used to study the entry mechanism of SARS-CoV-2 as well as potential treatments and vaccines (Han et al., 2021; Spitalieri et al., 2022). In fact, human lung organoids (hLORGs) have been demonstrated to be very important models that can predict the infection outcome and can be also used as a drug screening platform to foster research for new efficient therapies. Numerous studies have confirmed that SARS-CoV-2 infection begins in the proximal airways or in alveolar type 2 (AT2) cells of the distal lung, which express both ACE2 and DPP4 receptors. Although common 2D human cell cultures are permissive to infection *in vitro,* they cannot faithfully reproduce the human pathophysiology (Duan et al., 2021). hLORGs have been validated instead to be useful *in vitro* models to simulate infections and screen new compounds. In fact, they express both ACE2 and DPP4 receptors, thus being permissive to SARS-CoV-2 entry and replication, as our data show (Spitalieri et al., 2022).

Therefore, hiPSC-derived organoids are important models that can be used to study the pathogenicity of infectious diseases, and alongside, they can be used as a platform for drug screening, speeding up the discovery of new therapies.

### 4.2 Genetic diseases

Organoids model very well human genetic diseases *in vitro*, as two different scenarios are easily reproducible: on one hand, they can be derived directly from the patient’s own cells, thus retaining any pathological mutations and variation, on the other hand, organoids derived from healthy cells can be genetically modified to reproduce a specific disease or condition. For example, Lee and coworkers (Lee et al., 2009) derived peripheral neurons from hiPSCs of a patient with Familial Dysautonomia (FD), a rare inherited sensory and autonomic neuropathy caused by point mutation of the *IKBKAP* gene. Due to the lack of an appropriate model system, the mechanism of neuron loss in this disease is not fully understood. Within their study, using hiPSCs-derived neural crest precursor cells, they were able to study the pathogenesis *in vitro* as well as possible treatments for this rare disease. Another well-established model for a genetic disease regards Cystic Fibrosis (CF). In fact, intestinal organoids have been extensively used to study CF, obtaining very promising results. An interesting application in this context is the use of intestinal CF model organoids to guide the decision-making process during clinical trials by pre-treatment stratification of *in vitro* responders Fare clic o toccare qui per immettere il testo. (van Mourik et al., 2018). De Winter-de Groot *et al.* in their study highlights the importance of using organoids in pre-clinical studies: they not only were able to characterize rare *CFTR* variants, but also they could make a prediction of individual response to drugs and thus shed the light on the rare individual disease phenotypes (de Winter-De Groot et al., 2018).

Another example of how 3D organoids can support the study of a genetic disease comes from the brain. Scientists have always been fascinated by the brain due to its complexity and the poor accessibility of its tissue which limited the research on its pathophysiology. The only non-invasive techniques that allowed to study the neurodevelopment were ultrasound imaging, animal models, or abortus material (Trujillo and Muotri, 2018). The advent of hiPSC-derived brain organoids has been an important turning point. Lancaster *et al.* reported the first protocol to generate cerebral organoids, which included interdependent brain regions, such as the cerebral cortex containing progenitor populations that produce mature cortical neuron subtypes (Lancaster et al., 2013). Later on, other studies successfully developed brain organoids containing other regions, such as the forebrain, cerebellum, cortex, hippocampus, midbrain, and hypothalamus. The ability to obtain the different structures allows not only to study specific conditions linked to a particular region, but also to study the interconnection between different cell subtypes during the developmental stages. One of the major applications of brain organoids is the study of Microcephaly, which is an autosomal-recessive condition caused by mutations in several genes. For example, the mutation of the *TREX1* gene causes Aicardi Goutieres Syndrome (AGS) type I, which is characterized by dramatic neuronal loss and leads to lifelong disability (Crow and Manel, 2015). Organoids are an important tool through which oit is possible to discriminate how AGS influences the functionality of the various subtypes of cells that compose the brain, thanks to the possibility to compare the disease model with an isogenic control (Trujillo and Muotri, 2018). In the context of genetic disease, it is fundamental to compare experiment results between isogenic conditions, thus hiPSC-derived organoids are a game-changer in this field.

### 4.3 Gene editing

**FIGURE 2:**
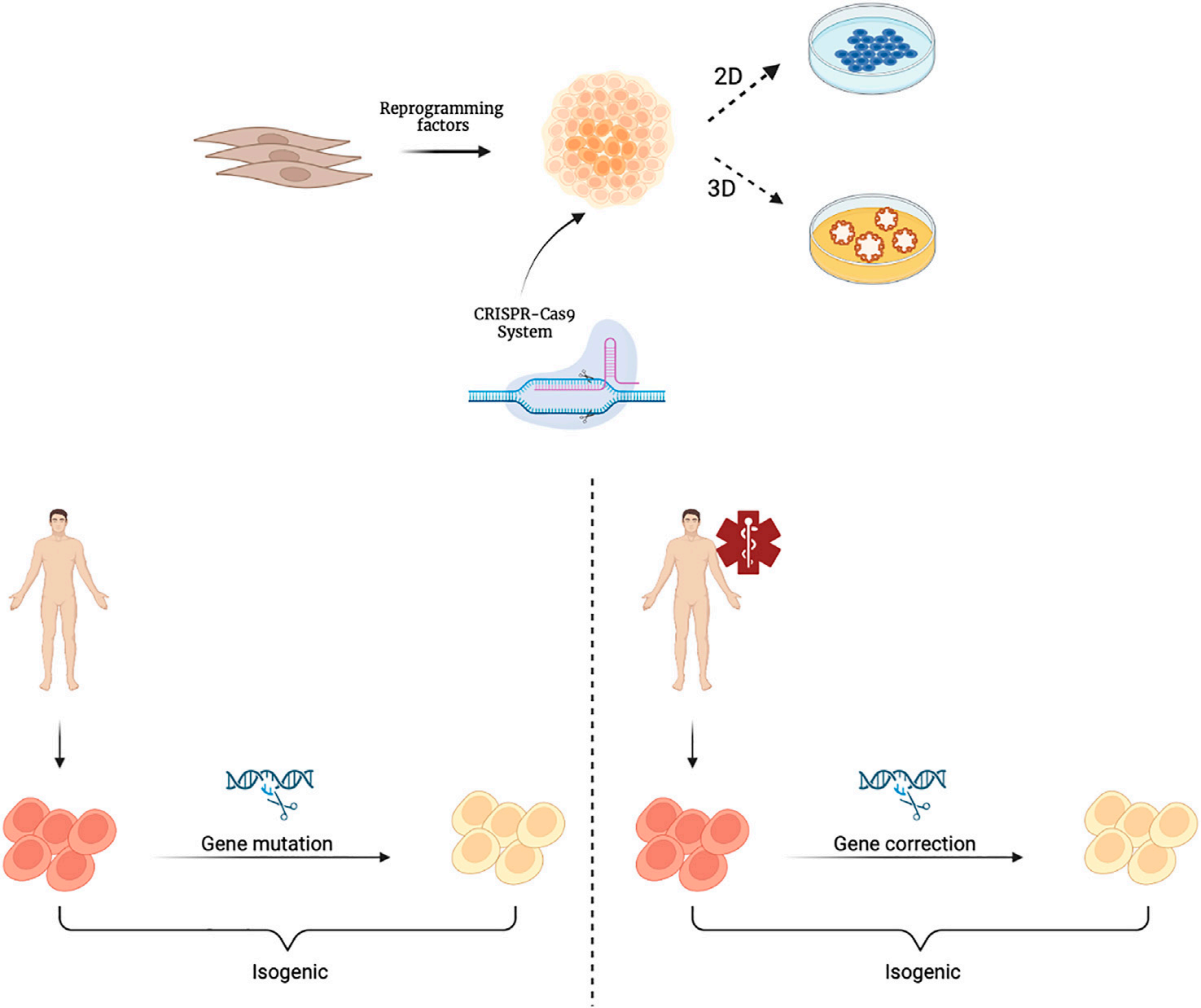
Schematic representation of hiPSCs derivation and their gene editing with the CRISPR-Cas9 system. CRISPR-Cas9 technology is applied to cultures of hiPSCsthat can be further differentiated and cultivated in 2D or 3D organoids. Isogenic hiPSCs or organoids can be obtained from healthy individuals through genetic mutations (left) or from patients through gene corrections (right). Created with BioRender.com.

A further step forward in the context of rare genetic diseases is the application of gene editing techniques, such as CRISPR (clustered regularly interspaced short palindromic repeats), which combined with protein 9 (Cas9)/CRISPR, makes it easy to target any genomic locus that can be recognized by a specific RNA guide (Artegiani and Clevers, 2018). The use of organoids as *in vitro* models is particularly useful in the context of rare genetic diseases (Anderson and Francis, 2018). A rare disease is defined as a disease whose prevalence, that is, the number of cases present at a given time in a given population, does not exceed 1 case per 2,000 population. It is a field in which it is difficult both to study pathophysiology, because few samples are available, and to evaluate the efficacy and safety of possible therapies, as there is not a large enough number of patients to draw statistical conclusions appropriate to standards. It is clear how, in this context, the use of organoids as *in vitro* models is particularly useful. In fact, the reproducibility of organoids as well as their ability to retain all of the patient’s genetic information is a valuable tool for the study of these diseases. In addition, through the use of CRISPR-Cas9 technology, the mutations responsible for these rare diseases could be corrected thus achieving isogenic control through which the genotype-phenotype correlation in different organoids representative of the various tissues involved in pathophysiology can be studied (Figure 2). For example, CRISPR-based editing was used to convert *SMN2* to an *SMN1*-like gene in hiPSCs derived from patients with Spinal Muscular Atrophy (SMA) caused by homozygous *SMN1* gene deletions. Subsequent differentiation into motor neurons provided proof of principle for this gene correction strategy in SMA (Lin et al., 2020). The ability of the organoids to be expanded for multiple passages offers the opportunity to establish modified lines for precise gene correction of the disease-causing mutation. Such technology can be advantageous as it can shed the light on the biology of essential genes.

Currently, some of the best-known applications of CRISPR-Cas9 technology on organoids include cancer organoid models (Hendriks et al., 2020), intestinal organoids for the study of Cystic Fibrosis (CF) (Geurts et al., 2021), and *DGAT1* gene regulation (Schene et al., 2020), kidney organoids for the study of Autosomal Dominant Polycystic Kidney Disease (ADPKD) (Freedman et al., 2015; Kuraoka et al., 2020), and brain organoids for the study of Trisomy 21 (Tang et al., 2021), the *CHD8* gene involved in Autism Spectrum Disorders (ASD) (Wang et al., 2017), and GM1 Gangliosidosis (Latour et al., 2019). The ability to engineer the genome of hiPSC/hORG cultures may provide new insights into cellular characteristics, differentiation, and disease mechanisms, while also giving evidence for the principle of gene repair.

### 4.4 Tumor models

The possibility of taking cells from a tumor and culturing them with appropriate protocols paved the way for the creation of the 3D “tumoroids”, which have enormous advantages over 2D cultures that fail to reproduce the tumor tissue microenvironment (TME) (Jensen and Teng, 2020). In fact, one of the difficulties while making a diagnosis and then during the decision on the most appropriate cancer treatment is the intra and inter-tumor heterogeneity. In addition, tumor development is highly dynamic, due to chromosome and microsatellite instability, and epigenetic modifications (Xia et al., 2019). Tumoroids are 3D self-assembling organoids, which derive directly from the patient tumor cells, thus including various cell subpopulations with different gene alterations and managing to maintain the cellular heterogeneity that makes up the tumor *in vivo* and the genetic signature of the host patient. Because of these characteristics, they are considered a predictive platform for patient response to the range of existing treatments, guiding the decision-making process and clinical studies in a more effective and efficient way (Zhou et al., 2021b). Since they were first established, tumoroids gained popularity and to date, there are more than ten kinds of cancer models of patient-derived organoids (PDOs).

**FIGURE 3:**
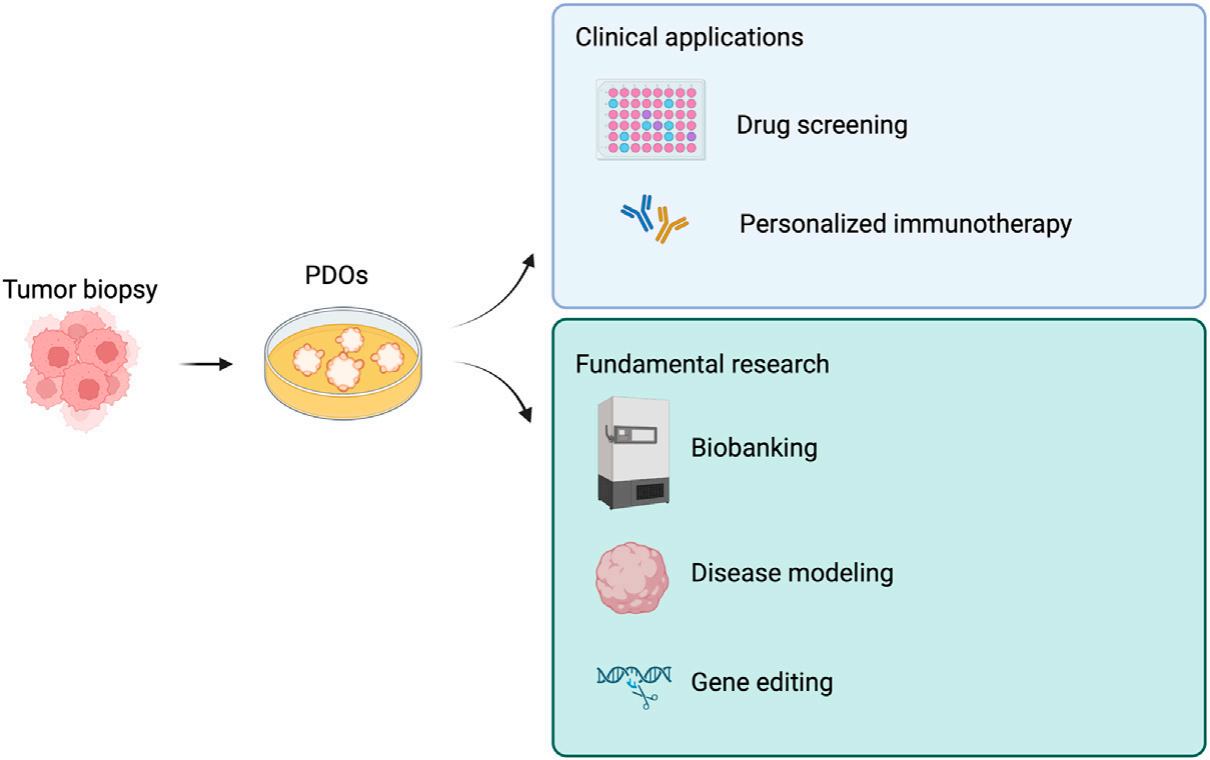
Derivations and potential applications of patient-derived tumor organoids (PDOs). PDOs can be used in the clinic as a drug screening platform and to guide personalized immunotherapy. Similarly, PDOs are a useful tool for fundamental research. Created with BioRender.com.

We summarized tumoroid applications in Figure 3, while in Table 2 we aimed at highlighting few examples of how versatile tumoroids are, offering a comprehensive overview of the recent advances in this field.

**TABLE 2:** Overview of the major advances and applications in patient-derived tumoroids.

Tumor model	3D culture	Microfluidic device	Notes	References
Lung	✓		Identification of therapeutic targets based on NGS results of patient-derived organoids	Yokota et al. (2021)
Metastatic colorectal cancer	✓		Test on drug sensitivity and resistance using a gel-free 3D cutlure device	Sogawa et al. (2021)
Triple-negative breast cancer		✓	Multi-parametric profiling of drug sensitivity on individual tumor types	Cromwell et al. (2022)
Cervical tissue	✓		Endo- and ectocervical derived organoids that can recapitulate *in vitro* the viral infections (e.g. HPV) cause of the cervival cancer	Lõ et al. (2021)
Liver	✓		Bio-active silk 3D tunable matrix that enable prolonged growth, high metabolic activity and overall enhancement of tumor properties	Arora et al. (2021)
Glioblastoma	✓		Optimization of defined media and protocol for culturing glioblastoma organoids, used to model personalized CAR-T immunotherapy	Jacob et al. (2020)
Pancreas	✓		Tumoroid profiling, using a multi-omics approach, to identify molecular and functional cancer subtypes to predict therapeutic responses	Tiriac et al. (2018)
Rectal cancer	✓		Living biobank of 80 patient-derived rectal cancer tumoroids. They demonstrated to accurately confirm the clinical outcomes of locally advanced rectal cancer (LARC)	Yao et al. (2020)

Although tumoroids advocate contributing to the field of precision medicine by establishing a high throughput drug screening platform, currently there are still some challenges that need to be addressed. For example, to date, tumoroids cannot fully replicate the tumor tissue microenvironment, which is usually composed of a complex interconnection of blood vessels, immune cells, and neurons. These different types of cells influence in different ways cell proliferation and extracellular matrix production (Orimo et al., 2005). Thus, having a co-culture of tumor cells together with fibroblasts and immunocytes could help achieve a higher fidelity platform. Quéméneur and others, indeed, successfully included fibroblasts and macrophages in a patient-derived tumoroid observing an enrichment in immune cells and obtaining the expression of the markers of lung adenocarcinoma (Lê et al., 2022). These flourishing examples are paving the way for the use of tumoroid cultures in clinical trials as well as for basic research.

### 4.5 Toxicology testing

Toxicology is one of the fields in which organoid technology can be better explored and used. For their characteristic of 3D miniaturizations of human tissues, the organoids exhibit native tissue architecture (Clevers H.et al., 2016) that carries out person-specific genomic and epigenetic information. Although 2D cultures are associated with simple and low-cost maintenance of the cell culture, in adherent cultures cell-cell and cell-extracellular environment interactions cannot be represented. These interactions are responsible for cell differentiation, proliferation, vitality, expression of genes and proteins, responsiveness to stimuli, drug metabolism, and other cellular functions (Mseka et al., 2007; Weaver et al., 2002; Meyers et al., 2003). Another drawback of 2D systems is that cells cultured in a monolayer have unlimited access to the ingredients of the medium such as oxygen, nutrients, metabolites, and signal molecules. Furthermore, it has been observed that the 2D system changes the gene expression and splicing, topology, and biochemistry of the cell (Birgersdotter et al., 2005; Li et al*.,* 2006). In addition, adherent cultures are usually monocultures and allow for the study of only 1 cell type.

Also, the absence of relevant animal models for many human diseases, as well as the inaccurate prognosis coming from ‘conventional’ pre-clinical models, are among the major reasons for the failures observed in clinical trials. In addition, there is a need to confirm drug safety in both male and female humans (Soldin and Mattison, 2009) along with high-throughput screening of a wide range of drugs and compounds for different purposes, including the development of novel contraceptive agents and vaccines, drug screening for infectious diseases, cancer drug development, gestational drug development and reproductive toxicity testing of drugs and compounds (Chapman et al., 2013; Brannen et al., 2017).

Recently, a study on endometrial cancer organoids was published in which patient-specific drug responses was reported. A specific endometrial cancer organoid line was most sensitive to *everolimus* (an inhibitor of mammalian target of rapamycin mTOR), which suggested a strong dependence on the PI3K-AKT pathway and was in line with mutations in the pathway’s signaling mediators (PTEN, PIK3CA, AKT1) (Borretto et al., 2019).

Also, Price et al. reported a suspension technique for efficient large-scale cancer organoid culturing. In this study, they tested the sensitivity of organoid models to 72 anti-cancer drugs from three different cancer types, monitoring global and disease-relevant molecular changes and phenotypes including drug sensitivity and genetic dependencies (Price et al., 2022).

An important example of a patient-specific efficacy drug test, conducted in the laboratory, was reported by Dekkers and others (Dekkers et al., 2016). Indeed, it is well known that Cystic Fibrosis drugs modulate the function of the CFTR protein, but the drug’s effectiveness depends on which CFTR mutation a patient carry. The authors showed that drug responses observed in mini-guts or rectal organoids can be used to predict which patients may be potential drug responders. This preclinical test can help quickly to identify responders to CFTR modulating drug therapy even when patients carry very rare CFTR mutations. Also, a more recent study by Berkers (Bekers and van Mourik, 2019) demonstrates that *in vitro* drug responses in rectal organoids from individual patients with Cystic Fibrosis correlate with changes in two *in vivo* therapeutic endpoints. These results indicate that an *in vitro* assay using stem cell cultures can prospectively select efficacious treatments for patients and suggests that biobanked stem cell resources can be used to tailor individual treatments in a cost-effective and patient-friendly manner.

Finally, with the purpose of testing drug toxicity, more and more studies are exploring the benefits of putting together two important technologies in this field: 3D organoids and microfluidic devices. The so-called organs-on-chip allow high sensitivity and reproducibility with respect to the other *in vitro* models, making the simulation of cellular response closer to the human *in vivo* counterpart while speeding up the time for the analysis. Hence, their usage can be perfectly integrated in the context of innovative tools for studying the interactions between organs and drugs, and consequently their safety and toxicity.

Every year, the process of drug development is slowed down due to the difficulty of predicting the kind and frequency of side effects that will manifest *in vivo* during the clinical phase. Eventually, adverse events in humans stop this long pipeline with consequent loss of time, money and resources. Therefore, breakthrough technologies are needed to cope with this hurdle (Augustyniak J. et al., 2019). Organs-on-a-chip not only have the ability to recreate the structural and functional features of human organs as other 3D models, but they also allow a fine control of the microenvironment by managing chambers microfluidics (Zhan, B. et al., 2018; Huh D. et al., 2010). Several parameters are easily accessible and supervised, such as fluid flow, cell–cell and cell–matrix interactions and, size and shape of the engineered organs. Within all the organs, the lung is the most reproduced organ-on-a-chip. Zhang et al. developed a novel 3D human lung-on-a-chip with which they assessed pulmonary toxicity of TiO2 and ZnO nanoparticles (Zhang M.et al., 2018).

Another example is the gut-on-a-chip investigated from Kim et al., who demonstrated with this model that gut microbiome, inflammatory cells and peristaltic contractility can deregulate intestine bacteria proliferation and inflammatory phenomena (Kim H.J. et al., 2016) Furthermore, progresses have been made on the development of a more and more accurate representation of the tumor tissue microenvironment (TME), which is essential in toxicity evaluation of new anti-cancer compounds. Indeed, Shirure et al. created a microfluidic system that simulates the *in vivo* vascular component of TME (Shirure V.S et al., 2018). The model reproduced perfusable blood vessels (Kim et al., 2013; Hasan A et al., 2015; Wang et al., 2016; Chen M.B. et al., 2017), thus addressing the need of giving the right amount of nutrient supply and expanding the life span with respect to standard organoid models. These are really important features when modeling the TME and consequently for having consistent results within the downstream analyses. For example, with the model described above, they were able to monitor different characteristics of tumor progression, like cell proliferation and the formation of new blood vessels. Additionally, these vascularized tumoroids could be cultured for over 22 days, allowing to assess the toxicological effects caused by vascular perfusion with paclitaxel, suggesting the advantageous use of this combined platform as preclinical patient-derived model to analyze responses to chemotherapy.

Wang et al. developed a brain organoid to model the pathological features (e.g., abnormal cortical development) occurring under prenatal environmental exposure to toxins such as nicotine (Wang Y. et al., 2018). Across a different study, he also developed a BBB microfluidic system created by derivatizing brain microvascular endothelial cells from iPSCs, allowing for the research of drug permeability and toxicity (caffeine, cimetidine, and doxorubicin).

Moreover, a higher level of complexity is pursued by the Sung group, pioneer in this field, that aim to combine different organoids and organoids-on-a-chip in order to get even closer to the reproduction of the human body, also called “human-on-a-chip”, with the purpose of developing a comprehensive model that could accurately predict interactions between organs and tissues under physiological or pathophysiological conditions (Sung et al., 2014; Skardal A. et al., 2016).

Finally, an innovative technology that usually plays an important role in this field and it is worth mentioning is the 3D bioprinting. It is greatly involved for the development of organoids or organoids-on-chip due to the ability to organize the cytoarchitecture the organoid layer-by-layer, allowing for a fine tune of the structural characteristics *in vitro*. Successful examples use the 3D printing for both the fabrication of the microfluidic device (Yazdi A.A. et al., 2016) and for the bioprinting of 3D tissues and organs (Ma J. et al., 2018) resulting in a synergistic combination of two independent technologies that allows the construction of systems that replicate the *in vivo* environment and may be widely employed for drug development and toxicity tests (Peng W. et al., 2017; Ma X. et al., 2018; Radhakrishnan J et al., 2020).

## 5 Organoid factories

In recent years, local reference centers for the cultivation and commercialization of organoids have sprung up around the world. This is an increasing trend that shed the light on the need of bridging the public sector (research institutes and universities) and the private sector. For example, a company that wants to simultaneously test a large number of drugs and new molecules has the possibility to collaborate with centers of excellence specialized in appropriate and already validated organoid models, to speed up the process of selecting the most promising drug formulations.

**FIGURE 4:**
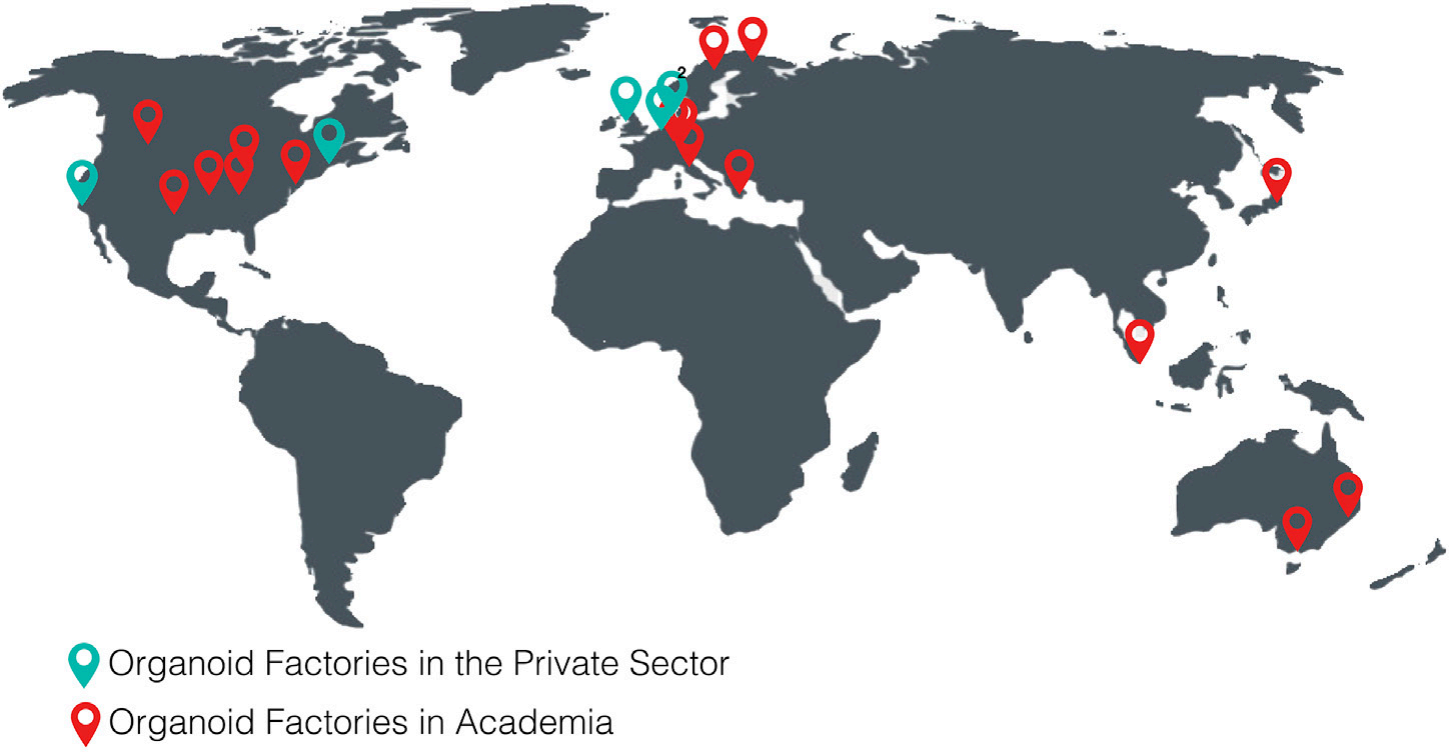
World distribution map of the organoid factories in academia and in the private sectorTables.

The following tables show the research institutes (Table 3) and companies (Table 4) specialized in 3D organoid cultures, also schematized in Figure 4.

**TABLE 3:** Research institutes around the world that have a Research and Development (R&D) laboratory dedicated to organoid cultures.

Name of the institute	Location	Kind of organoids	References
Cold Spring Harbor Laboratory	United States (New York)	Tumoroids	CSHL’s new organoid facility, (2022)
Karolinska Institutet Stem Cell Organoid	Sweden	hESCs and hiPSCs characterization and differentiation in organoids of interest for studying the developmental stage and diseases	KISCO, (2022)
University Of Colorado - Gates Center For Regenerative Medicine	United States (Colorado)	iPSC e ASC-derived organoids	Gates Center, (2022)
Tampere University - Adult Stem Cell Organoids Facility	Finland	Gut and esophagus organoids, derived from human and murine biopsies; Murine liver, cornea, kidneys, and prostate organoids	Adult Stem Cell Organoids Facility, (2022)
Cincinnati Children’s Hospital Medical Center - Custom	United States (Ohio)	Gut, liver, stomach, and colon organoids	Cincinnatichildren, (2022)
Max Planck Institute Of Molecular Cell Biology And Genetics - OSCF	Germany	Developmental studies on placenta and blastocyst, gastruloids; fibroblast differentiation into brain organoids, neuronal precursors, motoneurons; pancreas and liver spheroids	MPI-CBG, (2022)
Monash Biomedicine Discovery Institute	Australia	n.r	Organoid Program, (2022)
Istituto Nazionale Di Genetica Molecolare - Center For Engineered Multilineage Organoids In 3D Microenvironment	Italy (Milan)	n.r	Organoid Center, (2022)
University Of Chicago - OPCR	United States (Illinois)	The aim is to ease the access, development, creation, and use of organoids and primary cell lines derived from patients	(Organoid and Primary Culture Research Core, 2022)
Children’s Medical Research Institute	Australia	iPSC derivation, cultivation, and differentiation into organoids (brain, cortical and cerebral, heart, kidneys)	CMRI, (2020)
Max Delbrück Center (Mdc)	Germany	Engineered brain organoid cultures	Organoids, (2022a)
University Of Calgary - Snyder Institute For Chronic Diseases - Human Organoid Innovation Hub	Canada	Human and murine cell-derived gut and colon organoids	Human Organoid Innovation Hub (2022)
Forth - Biomedical Research Institute - Human Brain Organoids Facility	Greece	hiPSC and hESC-derived brain organoids to study the developmental stage	(Human Brain Organoids Facility)
National University of Singapore - Induced Pluripotent Stem Cells (Ips) & Organoid Central Facility	Singapore	n.r	Life Sciences Institute, (2022)
Human Organoid Project	Japan	n.r	Miraikan The National Museum of Emerging Science and Innovation, (2022)
Washington University In St.Louis - PAMOC	United States (Missouri)	Gastrointestinal organoids are derived from human and murine cells	Precision Animal Models and Organoids Core (2022)

n.r. not reported.

**TABLE 4:** Companies that have an R&D department on organoid cultures.

Company name	Location	Applications	References
Epistem	United Kingdom	Liver and gut organoid models	Epistem, (2022)
CorEuStem: The European Network for Stem Cell Core Facilities	Belgium	n.r	Action CA 20140, 2022
Beth Israel Deaconess Medical Center	United States (Massachusetts)	Patient-derived tumor organoids	Organoid Core, (2022)
Organoid Innovation Center	United States (California)	3D cell culture system, modular and automated, that allows to acquire and elaborate images and makes the drug discovery process scalable and reproducible	Molecular Devices, (2022)
HUB	Netherlands	Disease modeling, R&D on the development of new drugs, Clinical Trials in a Dish (CTiD), toxicity and safety tests, personalized medicine	HUB Organoids, (2022)
InnoSer	Belgium	Brain organoids and tumoroids	Organoids, (2022b)

n.r. not reported.

## 6 Discussion

In recent years, organoids have often been considered the technological solution that could overcome the obstacles found in the use of current 2D *gold standards* and their applicability in the drug screening process. Despite this, to date, these versatile 3D cultures by their very nature have features that limit their applications. Nevertheless, researchers worldwide are working in the direction of creating more and more accurate and useful models.

Here below we discuss some of the trending topics that are rising among the development and the applicability of organoids.

### 6.1 Maturity

According to transcriptional and functional analyses, hiPSCs-derived organoids typically resemble a fetal-like stage and cells are indeed partially immature. On the one hand, this is a disadvantage when studying mechanisms that involve adult tissue. However, it is possible to force organoid maturation by adding complex factors to the culture media or by inducing overexpression of aging genes (Miller et al., 2013; Ho et al., 2016). Other studies use biophysical stimulation *via* electromechanical inputs instead of achieving functional maturation of hiPSCs-derived cardiomyocytes (Ruan et al., 2016; Ronaldson-Bouchard et al., 2019). On the other hand, the lack of maturity could be an advantage when studying late-onset disease with a genetic component, which could have a great influence on cells during development. For example, the number of CAG repeats has a positive correlation between the age of onset of symptoms and the severity of the disease. Studying these diseases through organoids could lead to new insights into disease progression. In fact by identifying and using prophylactic drug treatments to be administered early in the lives of at-risk patients, it could be possible to correct the biological dysfunction and cure or limit the disease even before its onset. An example is the use of statins that are administered years in advance of the onset of heart disease to lower cholesterol and prevent Atherosclerosis. Another example is Huntington’s Disease (HD), a neurodegenerative disease in which the age-of-onset of symptoms has a positive correlation with the number of CAG repeats in the first exon of the *Huntingtin* gene (Andrew et al., 1993). This leads to the production of a mutant protein (mtHTT) with an expanded polyglutamine repeat (polyQ). To date, little is known about how this protein causes neuronal loss of functions and toxicity. Garcia *et al.* (Garcia et al., 2019) developed a protocol to obtain an iPSC-based HD model to study Huntington’s Disease. In particular, they obtain functionally mature astrocytes in co-culture with neuronal cells, that resemble phenotypes previously seen in HD mouse models, such as impairment of Inward-rectifier potassium channels (K_ir_) currents, lengthened spontaneous Ca^2+^ waves, and reduced cell membrane capacitance. The ability to faithfully reproduce the *in vivo* conditions is leading to new findings on HD and the mechanistic pathways, paving the way for new drug targets.

In conclusion, models established from patient-derived hiPSCs, even with their current immaturity, provide relevant information about pathophysiology that can guide the development of new treatments and clinical decisions.

### 6.2 Organoid interconnections

Another important aspect is the lack of vascular and immunological components in organoids, which are typical instead of an explant organ. The direction in which research in this field is heading is the development of *assembloids*: manually forcing the union of organoids or fusing different cell types so as to unite multiple systems in one platform, possibly using morphogenic gradients as “organizers” of substructures (Pasca, 2019). Examples of assembloids are the combination of organoids resembling different regions of the brain, such as dorsal and ventral forebrain, or thalamic and cortical organoids to study the spatial organization and functional interaction between the various subtypes of cells (Xiang et al., 2019). The role of assembloids is particularly important when studying complex conditions such as autoimmune diseases (e.g. multiple sclerosis) which affects the immune system, as well as the nervous system. Thus, having both components in the same model can shed the light on their interplay interactions.

Furthermore, Lin *et al.* (Lin et al., 2018) developed multi-lineage hiPSC-derived assembloids to study neural-glial interaction in Alzheimer’s Disease. More technological solutions involve the use of tools at the microscale, such as miniaturized blood vessels that could be assembled with organoids of other types. These miniaturized blood vessels, in particular, contain endothelial cells and pericytes derived from hiPSCs and have been able to model mechanisms of diabetic vasculopathy (Wimmer et al., 2019). Microfluidic systems could instead facilitate gaseous and nutrient exchanges within the organoid. Other examples of assembloids include all those models aimed at studying the interaction between multiple organoids. For example, Workman *et al.* are working on intestinal organoids derived from hiPSCs that can be cultured with neural crest cells to recapitulate the development of the intestinal enteric nervous system and study motility disorders of the gastrointestinal tract (Workman et al., 2017). Last but not least, hiPSC-derived assembloids are very useful to study how chambers interact during development in organs that are composed of multiple compartments or cavities. Heart assembloids indeed offer insights into the interaction between atria and ventricles during the developmental stage (Sharma et al., 2020).

### 6.3 Reproducibility and standardization

The last critical issue related to cell culture, whether 2D or 3D, is the intensive demand for human resources (often even on weekends or holidays). The daily tasks are manual and repetitive, taking time away from equally important research activities such as studying, planning, and developing new protocols and experiments. The application of automation in cell culture laboratories would address the need to optimize the use of human resources, as well as cell culture processes, in order to achieve more accurate, reproducible, and scalable results.

Special attention should also be paid to process traceability, as well as to reducing the risk of contamination and the safety of operators who would no longer be handling hazardous chemicals. A successful example comes from Tristan *et al.*, who integrated a robotic platform to automate the basic processes of iPSCs cultivation and differentiation, giving evidence that automation allows rapid and standardized cell manufacturing thus increasing the efficiency for potential scalable applications, such as drug screening, and cell therapy (Tristan et al., 2021). Furthermore, there are already some companies that are specialized in helping cell culture laboratories to automate their workflow (Daniszewski et al., 2018).

In conclusion, automation could be a concrete way to accelerate the translation of research from the laboratory to the clinic.

### 6.4 Training

Organoid cultures require staff that is highly qualified, dedicated, and continuously updated on the latest available protocols (continuous staff training). For this reason, organoid factories around the world are fundamental reference centers for 3D cultures for all those laboratories and research institutes that need to use this technology to increase the efficiency of their drug screening tests but lack the facilities and specific knowledge on 3D cultures.

### 6.5 Costs

The high cost of producing and maintaining organoids certainly falls into one of the main disadvantages of using this technology. However, as Bose *et al.* (Bose et al., 2021) report, when compared with those required for animal experiments, organoids are profitable. In fact, for the aforementioned reasons of the higher efficiency of 3D models, their self-renewing ability, and the possibility of testing a large number of drugs simultaneously, on a large-scale organoids will be definitely the most advantageous choice from an economic point of view.

### 6.6 Ethics

Ethical concerns are arising as we approach the creation of more and more complex organoid systems, such as assembloids. Hyun *et al.* (Hyun et al., 2020) identified five ethical issues for brain organoids that could eventually be applied also to all the other kinds of organoids. First of all, this research field is constantly evolving and as with every new technology, there is the need to adapt the current regulations and policies to define the new borders to assure the ethics, safety, and objectivity of the research. Secondly, the development of organoids involves the procurement of biological materials. For iPSC-derived organoids, the human sample is simply composed of the donor’s somatic cells. It is important that the donor signs an informed consent, in which the risks and benefits of the patients are clear as well as the aims of the research study. The latest guidance for stem cell research can be found in the ISSCR Guidelines for Stem Cell Research and Clinical Translation (Lovell-Badge et al., 2021). Moreover, it is important that during the recruitment phase, patients are aware of the fact that organoid research is far away to be translated into the clinic.

The advantages of these studies will not directly involve the patient or any other relative. Furthermore, even if organoids have the aim of reducing the number of animals used for scientific purposes, it is important to state that to date we cannot get rid of pre-clinical animal tests, as regulations still require them. Lastly, the major ethical concern specifically regards brain organoids, as it involves the concept of “consciousness”. Scientists and ethicists are questioning whether even the most advanced model of brain organoid could eventually “be conscious”, even if the definition of consciousness is still very ambiguous. Alysson Muotri’s group (Trujillo et al., 2019) developed a cortical organoid model in which groups of neurons were functioning in unison, producing gamma, alpha, and delta waves. Although these patterns could be linked to the electrophysiological events registered in 25–39-week-old premature infants’ electroencephalograms (EEGs), it is still unclear whether this could mean having consciousness, rather than just having basic brain mechanics that would then be ethically innocuous.

## 7 Conclusion

To conclude, over the past decades, 3D-cultured organoids have rapidly gained popularity. This trend can be observed from the increasing number of research institutes and companies that have recently included a core facility or department exclusively focused on 3D cultures and stem cells. The advent of organoid factories around the world will result in the enhancement of this technology and will speed up the translation of this research from the bench to the bedside.

The hiPSC-derived organoid technology is now passing from a proof-of-principle to real applications in the clinic. This transition has been made possible also thanks to its versatility, given by the applicability of different techniques, such as CRISPR/Cas9 genome editing system, material engineering for the scaffolds, or microfluidic systems. The benefits of the development of accurate and validated organoid models will have a crucial role in the advance of both basic biological and translational research, particularly in the pharmacological field and drug development.

In fact, in the near future, 3D organoids will guide the clinical decision-making process, having validated patient-specific drug screening platforms. This is particularly important in the context of rare and/or undiagnosed genetic diseases or when testing cancer treatments that could in principle have severe side effects. Therefore, this technology has enabled the advancement of personalized medicine in a way never seen before.

However, there are still some limitations that need to be addressed, especially regarding the cost and time-consuming cell cultures and the interconnection of different organoids to create an even more complex model that could eventually recapitulate the systemic response of the organism.

Overcoming these challenges will be a step-change in the field of drug discovery and screening, catalyzing the advent of personalized medicine.
